# Latent profile analysis and influencing factors of kinesiophobia among young and middle-aged patients with coronary heart disease

**DOI:** 10.3389/fmed.2026.1795966

**Published:** 2026-05-13

**Authors:** Mengying Yang, Xunying He, Yi Hu, Yingying Zheng, Hui Zhang, Peipei Yu

**Affiliations:** 1 Department of Cardiovascular Medicine, The Central Hospital of Wuhan, Tongji Medical College, Huazhong University of Science and Technology, Wuhan, China; 2 Key Laboratory for Molecular Diagnosis of Hubei Province, The Central Hospital of Wuhan, Tongji Medical College, Huazhong University of Science and Technology, Wuhan, China

**Keywords:** coronary heart disease, influencing factors, kinesiophobia, latent profile analysis, young and middle-aged patients

## Abstract

**Objective:**

To explore latent profiles and influencing factors of kinesiophobia among young and middle-aged patients with coronary heart disease (CHD).

**Methods:**

This cross-sectional study employed convenience sampling to recruit young and middle-aged patients with CHD from a tertiary hospital in Wuhan between September and November 2025. Participants were investigated using a general information questionnaire, the Fear of Activity in Patients with Coronary Artery Disease (Fact-CAD), Exercise Self-Efficacy Scale (ESES), Social Support Scale for Exercise (SSSE), Generalized Anxiety Disorder-7 (GAD-7), and Piper Fatigue Scale-12 (PFS-12). Potential categories of kinesiophobia in patients were identified using latent profile analysis (LPA), and univariate analysis and multinomial logistic regression analysis were employed to investigate the influencing factors of different profiles.

**Results:**

LPA identified three distinct categories of kinesiophobia: low kinesiophobia-selective sensitivity type, moderate kinesiophobia-perceptual sensitivity type, high kinesiophobia-fear avoidance type. Multinomial logistic regression analysis revealed that educational attainment, number of stents implanted, exercise self-efficacy, exercise social support, and fatigue were significant factors influencing these categories (*p* < 0.05).

**Conclusion:**

Significant heterogeneity exists in kinesiophobia levels among young and middle-aged patients with CHD. Healthcare providers should focus on the distinct kinesiophobia characteristics of different patients and implement targeted intervention strategies to reduce kinesiophobia levels.

## 1 Introduction

Coronary heart disease (CHD, also universally defined as ischaemic heart disease, IHD in global epidemiological statistics), characterized by atherosclerotic lesions leading to coronary artery stenosis or occlusion and subsequent myocardial ischemia, is the leading cause of death globally. According to the 2025 Heart Disease and Stroke Statistics Update from the American Heart Association, there were approximately 254 million people living with IHD globally in 2021, with 8.99 million IHD-related deaths reported in the same year (1), representing 14.8% of all deaths worldwide (2). As the world’s largest developing country, China is facing a severe and growing challenge in the prevention and control of CHD. The China Cardiovascular Disease and Risk Factors Surveillance Program, a nationally representative survey conducted across 262 surveillance sites in 31 provinces, autonomous regions, and municipalities directly under the Central Government of China from 2020 to 2022, released preliminary findings that the crude prevalence rate of CHD among Chinese residents aged 18 years and older was 758 per 100,000 population (3). According to the statistics from 2022, the crude mortality rate of CHD was 135.08 per 100,000 population among urban residents in China in 2021, and 148.19 per 100,000 population among rural residents, which imposes a substantial public health and socioeconomic burden across the whole country (4).

Notably, the incidence and mortality rates of CHD among young and middle-aged adults aged 18–59 years have exhibited a consistent upward trajectory (5). Factors such as heightened social pressures, irregular work schedules, and insufficient physical activity in this demographic have driven the continuous rise in its incidence and mortality compared with earlier periods (6). As the primary income earners for families and core contributors to social development, this population not only faces heavy household economic burdens after CHD onset, but also brings huge potential loss to social productivity, highlighting the urgent need to address the unmet clinical needs of this specific group.

Cardiac rehabilitation, as a therapeutic intervention, has been proven to help slow the progression of atherosclerosis in patients with CHD, reduce their readmission rates and mortality, and improve their prognosis and quality of life. Exercise rehabilitation is the core component of cardiac rehabilitation (7, 8). Kinesiophobia, defined as an excessive and irrational fear or avoidance of physical activity due to concerns about bodily harm, has been reported in previous studies to occur in 20 to 87.20% of patients with CHD (9, 10). Kinesiophobia can result in decreased adherence to exercise rehabilitation programs among patients with CHD, thereby negatively impacting the progression and outcomes of cardiac rehabilitation (11, 12). Notably, existing research on CHD-related kinesiophobia has predominantly focused on elderly patients, with limited attention paid to young and middle-aged patients. Previous research has shown that kinesiophobia among young and middle-aged patients with CHD is at a moderate level (13). Compared with the elderly, young and middle-aged patients bear varying degrees of family and social pressures. They are worried that exercise might cause harm to their hearts, affecting their work and life. Therefore, they are reluctant or seldom engage in physical activities. More importantly, even the few studies targeting young and middle-aged patients with CHD primarily utilize the aggregate score from kinesiophobia scales to evaluate overall kinesiophobia levels, neglecting the inter-individual heterogeneity of kinesiophobia within this population. Latent profile analysis (LPA), which employs probabilistic models to fit and compare sample characteristics, facilitates the identification of distinct categories and is extensively used in psychology, medicine, and other disciplines. Consequently, this study targeted young and middle-aged patients with CHD, aiming to identify latent categories of kinesiophobia through LPA. Furthermore, it examines the influencing factors within each category, offering a foundation for the development of precise, personalized intervention strategies tailored to different patient subgroups.

## 2 Method

### 2.1 Design and participants

From September to November 2025, a cross-sectional survey was conducted at the Central Hospital of Wuhan using convenience sampling. Inclusion criteria: ① Aged 18–59 years; ② Patients diagnosed with CHD via coronary angiography; ③ CHD duration ≥ 3 months; ④ Being conscious, without communication barriers, and possessing adequate comprehension and expression abilities; ⑤ Voluntarily participating in the study and signing an informed consent form. Exclusion criteria: ① Patients with concomitant severe heart failure, respiratory failure, malignant tumors, etc.; ② Patients with other diseases or conditions that significantly affect physical activity, including severe musculoskeletal diseases (such as advanced osteoarthritis, spinal cord injury, lower limb fracture within 6 months), severe neurological impairment (such as stroke with limb hemiplegia, Parkinson’s disease). This criterion was implemented through two steps: first, we reviewed the patient’s electronic medical records to confirm the diagnosis of the above-mentioned diseases; second, the attending physician of the patient was consulted to evaluate whether the disease would significantly limit the patient’s ability to perform daily physical activity and cardiac rehabilitation exercise. Patients with confirmed significant activity limitation were excluded.

This study was a cross-sectional investigation. According to cross-sectional sample size calculation methods, the sample size should be 5 to 10 times the number of variables. With 19 variables included in this study and accounting for a 20% non-response rate, the total sample size should be at least 100.

Before the study began, it was approved by the Ethics Committee of the Central Hospital of Wuhan, with the approval number: WHZXKYL2025-161.

### 2.2 Measures

#### 2.2.1 General information questionnaire

This questionnaire was designed by the researcher and included items on gender, age, educational attainment, marital status, living arrangements, monthly household income per capita, duration of illness, and heart function classification.

#### 2.2.2 Fear of activity in patients with coronary artery disease (Fact-CAD)

Fact-cad was compiled by Ozyemisci-Taskiran et al. (14). through literature review, semi-structured interviews, expert panel evaluation and pre-investigation, and was localized by Chinese scholar Hu (15). This scale is a single-dimensional one, consisting of 21 items, used to describe the patient’s daily activity ability. It adopts a 5-level scoring system, with scores ranging from 0 to 4 from “none” to “always.” Among them, 7 items are scored in reverse, and the higher the total score, the greater the degree of kinesiophobia. This scale has good reliability and validity among patients with CHD in Turkey and can be used as an assessment tool for the degree of kinesiophobia in patients with CHD.

#### 2.2.3 Exercise self-efficacy scale (ESES)

This scale was developed by Bandura (16) and localized and revised by Tung (17). It consists of 18 items, with a total score ranging from 0 to 100. The calculation method is to add up the scores of each item and then divide by the total number of items. The higher the score, the higher the self-efficacy in sports. In this study, the Cronbach coefficient of this scale was 0.953. The Cronbach ‘*α* coefficient of the Chinese version of ESES was 0.96, and the test–retest reliability was 0.861.

#### 2.2.4 Social support scale for exercise (SSSE)

The SSSE was developed by Zhong et al. through literature review, qualitative interviews, expert consultation, and questionnaire surveys (18). The scale comprises four dimensions with 24 items, employing a five-point Likert scale ranging from “1 = Strongly disagree” to “5 = Strongly agree.” It demonstrates sound structural validity, internal consistency, test–retest reliability, and criterion-related validity.

#### 2.2.5 Generalized anxiety disorder-7 (GAD-7)

GAD-7 is employed to assess participants’ emotional state over a two-week period (19). Comprising seven items, it utilizes a four-point Likert scale, with responses ranging from ‘never’ to ‘almost daily’ scored from 0 to 3 points, respectively. The maximum total score is 21 points, with scores of 5–9 indicating mild anxiety, 10–14 moderate anxiety, and 15–21 severe anxiety. The Cronbach’s *α* coefficient is 0.77.

#### 2.2.6 The Piper Fatigue Scale-12 (PFS-12)

The scale was developed and revised by Piper et al. (20), and translated into Chinese by Qiao et al. (21). The PFS-12 is a self-report questionnaire designed to assess patients’ current fatigue status, covering four dimensions: behavioral, emotional, sensory, and cognitive, comprising 12 items in total. It employs a 0–10 numerical rating scale, with all items scored positively. Scores are calculated by summing all item scores and dividing by the total number of items or the number of items within that dimension. Higher scores indicate more severe fatigue. The total scale reliability is 0.92.

### 2.3 Data collection

Before the commencement of the study, the investigators underwent standardized training and utilized consistent instructions to communicate the research’s purpose and significance to the participants. Following the acquisition of informed consent, questionnaires were distributed and collected on-site. Investigators were available to promptly address any participant inquiries during the questionnaire completion process. For participants unable to independently complete the questionnaire, investigators provided assistance through a question-and-answer format. Upon completion, the questionnaires were immediately reviewed for completeness, and any omissions were promptly returned to participants for supplementation. In total, 300 questionnaires were distributed, resulting in 281 valid responses, which corresponded to a valid response rate of 93.67%.

### 2.4 Statistical analysis

LPA was conducted using Mplus 8.3 software. Model selection was based on the Akaike Information Criterion (AIC), the Bayesian Information Criterion (BIC), the sample-size adjusted Bayesian Information Criterion (aBIC), the entropy value, the Bootstrapped Likelihood Ratio Test (BLRT), and the Lo–Mendell–Rubin Likelihood Ratio Test (LMRT). Lower values of AIC, BIC, and aBIC indicate better model fit, while entropy values closer to 1 reflect higher classification accuracy. *p* < 0.05 for BLRT or LMRT indicates that the k classes model fits better than the k-1 classes model. Data analysis was performed using SPSS 26.0 software. Normally distributed quantitative data were described as (x ± s), and intergroup comparisons were conducted using analysis of variance. Non-normally distributed quantitative data were described as *M* (P_25_, P_75_), and intergroup comparisons were performed using the Kruskal-Wallis test. Count data were described as frequency and percentage, and intergroup comparisons were conducted using the Chi-square test or Freeman–Halton test. The significance level was set at *α* = 0.05.

## 3 Results

### 3.1 Characteristics of participants

A total of 281 young and middle-aged patients with CHD were included in this study. The majority of patients were male (76.9%), with a mean age of 55 (49, 58) years. In terms of sociodemographic characteristics, most patients had an educational attainment of high school or vocational school (41.6%), lived with their families (87.2%), resided in city (47.7%), were married (87.9%), and had a family per capita monthly income concentrated at over 5,000 RMB (48.0%). Clinically, over 40 % of patients had a disease duration of 1–5 years (47.3%), more than half of the patients were classified as Grade I in heart function (56.9%), and approximately half (49.5%) had one or fewer stents implanted. Notably, patients who smoked and drank alcohol accounted for 38.1 and 47.0%, respectively.

### 3.2 LPA of kinesiophobia among young and middle-aged patients with CHD

LPA was conducted using Mplus 8.3, with the individual items of the kinesiophobia scale serving as manifest indicators for young and middle-aged patients with CHD. We sequentially explored models with one to four classes. The fit statistics are presented in Table 1. Across models 1 to 4, the Akaike Information Criterion (AIC), Bayesian Information Criterion (BIC), and adjusted BIC (aBIC) values continuously decreased as the number of classes increased. However, the Lo–Mendell–Rubin Adjusted LRT Test (LMRT) for the four-class model failed to reach statistical significance (*P* > 0.05), and the model lacked parsimony. After a comprehensive consideration of the model fit indices and the clinical interpretability of the classifications, the three-class model was ultimately selected as the optimal solution.

**Table 1:** Fitting results of a potential profile analysis model for kinesiophobia among young and middle-aged patients with CHD (*n* = 281).

Model	AIC	BIC	aBIC	Entropy	*P* (LMRT)	*P* (BLRT)	Latent profile probability
l	18762.111	18914.922	18781.741	—	—	—	1
2	17734.322	17967.177	17764.235	0.983	0.011	<0.001	0.16/0.84
3	17464.061	17776.959	17504.256	0.986	0.013	<0.001	0.13/0.41/0.46
4	17294.116	17687.058	17344.594	0.967	0.136	<0.001	0.08/0.28/0.09/0.54

AIC, Akaike Information Criterion; BIC, Bayesian Information Criterion; aBIC, sample-size adjusted Bayesian Information Criterion; LMRT, Lo-Mendell-Rubin Likelihood Ratio Test; BLRT, Bootstrapped Likelihood Ratio Test; —indicates no data available for this item.

### 3.3 Naming the latent profiles of kinesiophobia

**Figure 1:**
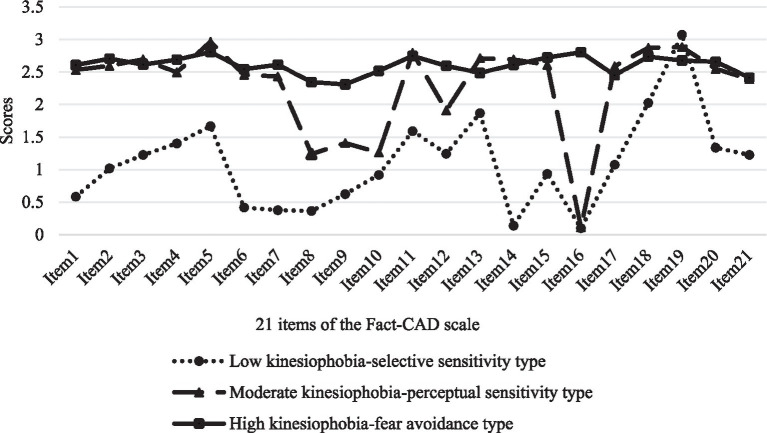
Latent profile characteristics of kinesiophobia among young and middle-aged patients with CHD. The specific information corresponding to each item can be found in Supplementary material 1.

The analysis ultimately yielded a three-class latent profiles based on the 21 manifest indicators (Figure 1). The profiles were then characterized and named according to the patterns observed in the line plots of the item means. Profile 1 was named the “low kinesiophobia-selective sensitivity type.” Patients in this group had overall score significantly lower than the other two profiles. However, they showed a distinctively elevated score on item 19 (“My heart problem may get worse if I stay inactive.”), indicating a selective sensitivity to the potential long-term health consequences of not exercising. Profile 2 was named the “moderate kinesiophobia-perceptual sensitivity type.” This group demonstrated moderate score across all dimensions, suggesting a certain degree of risk perception and moderate fear associated with exercise. Profile 3 was named the “high kinesiophobia-fear avoidance type.” Patients in this category had the highest overall score across all dimensions, exhibiting prominent kinesiophobia and fear-avoidance behaviors.

### 3.4 Univariate analysis of kinesiophobia profiles

Univariate analysis revealed that there were statistically significant differences among the profiles in terms of educational attainment, the number of stents, exercise self-efficacy, exercise social support and fatigue (*p* < 0.05) (See Table 2).

**Table 2:** Univariate analysis of kinesiophobia profiles among young and middle-aged patients with CHD (*n* = 281).

Variables	Low kinesiophobia-selective sensitivity type (*n* = 36)	Moderate kinesiophobia-perceptual sensitivity type (*n* = 115)	High kinesiophobia-fear avoidance type (*n* = 130)	Statistical value	*p*
Age	55 (45.25, 57.75)	55 (46, 57)	56 (50, 58)	3.829^3^	0.147
Gender					
Male	31 (86.1%)	89 (77.4%)	96 (73.8%)	2.415^1^	0.299
Female	5 (13.9%)	26 (22.6%)	34 (26.2%)		
Marital status					
Unmarried	3 (8.3%)	11 (9.6%)	6 (4.6%)	8.212^2^	0.071
Married	33 (91.7%)	94 (81.7%)	120 (92.3%)		
Divorce or widowed	0 (0.0%)	10 (8.7%)	4 (3.1%)		
Educational attainment					
Junior high school and below	8 (22.2%)	29 (25.2%)	50 (38.5%)	9.615^3^	**0.008**
High school or vocational school	14 (38.9%)	49 (42.6%)	54 (41.5%)		
Associate degree or higher	14 (38.9%)	37 (32.2%)	26 (20.0%)		
Residence situation					
Living alone	6 (16.7%)	15 (13.0%)	15 (11.5%)	0.673^1^	0.714
Living with families	30 (83.3%)	100 (87.0%)	115 (88.5%)		
Monthly per capita household income					
<2000	5 (13.9%)	21 (18.3%)	18 (13.8%)	0.505^3^	0.777
2000–5,000	12 (33.3%)	36 (31.3%)	54 (41.5%)		
>5,000	19 (52.8%)	58 (50.4%)	58 (44.6%)		
The location of the family					
Rural area	4 (11.1%)	17 (14.8%)	20 (15.4%)	0.883^1^	0.927
County town	15 (41.7%)	45 (39.1%)	46 (35.4%)		
City	17 (47.2%)	53 (46.1%)	64 (49.2%)		
Course of the disease					
<1 year	12 (33.3%)	32 (27.8%)	32 (24.6%)	3.688^3^	0.158
1–5 years	15 (41.7%)	41 (35.7%)	77 (59.2%)		
>5 years	9 (25.0%)	42 (36.5%)	21 (16.2%)		
The number of stents					
≤1	26 (72.2%)	63 (54.8%)	50 (38.5%)	15.002^3^	**0.001**
>1	10 (27.8%)	52 (45.2%)	80 (61.5%)		
Heart function classification					
I	22 (61.1%)	59 (51.3%)	79 (60.8%)	2.976^3^	0.226
II	13 (36.1%)	47 (40.9%)	45 (34.6%)		
III	1 (2.8%)	9 (7.8%)	6 (4.6%)		
Smoking					
Yes	14 (38.9%)	43 (37.4%)	50 (38.5%)	0.041^1^	0.980
No	22 (61.1%)	72 (62.6%)	80 (61.5%)		
Drinking alcohol					
Yes	15 (41.7%)	56 (48.7%)	61 (46.9%)	0.544^1^	0.762
No	21 (58.3%)	59 (51.3%)	69 (53.1%)		
Exercise self-efficacy	64.60 ± 15.96	53.00 ± 12.59	52.22 (38.89, 57.78)	26.056^3^	**0.000**
Exercise social support	80 (79, 83)	74 (70, 77)	70 (67, 71)	111.049^3^	**0.000**
Anxiety	4 (2.25, 5)	4 (4, 5)	4 (3, 5)	2.778^3^	0.249
Fatigue	3.20 ± 0.45	3.58 (3.33, 3.92)	4.08 (3.67, 4.33)	67.568^3^	**0.000**

(1) Chi-square test, (2) Freeman–Halton test, (3) Kruskal-Wallis test. Bold values indicate statistical significance (*p* < 0.05).

### 3.5 Multinomial logistic regression analysis of kinesiophobia profiles

A multinomial logistic regression analysis was performed with the latent kinesiophobia profile as the dependent variable, using the “low kinesiophobia-selective sensitivity type” as the reference category. Variables that were statistically significant in the univariate analysis were entered into the model as independent variables. The results indicated that educational attainment, the number of stents, exercise self-efficacy, exercise social support and fatigue were significant influencing factors for differentiating between the latent profiles (*p* < 0.05) (See Table 3).

**Table 3:** Multinomial logistic regression analysis of kinesiophobia profiles among young and middle-aged patients with CHD (*n* = 281).

Variables	Moderate kinesiophobia-perceptual sensitivity type				High kinesiophobia-fear avoidance type			
	*β*	Wald	*p*	OR	*β*	Wald	*p*	OR
Educational attainment								
Junior high school and below	1.010	1.706	0.191	2.744	1.968	5.267	**0.022**	7.159
High school or vocational school	0.149	0.053	0.818	1.160	0.559	0.584	0.445	1.748
The number of stents								
≤1	−1.470	5.498	**0.019**	0.230	−2.440	12.556	**0.000**	0.087
Exercise self-efficacy	−0.099	12.703	**0.000**	0.906	−0.133	20.057	**0.000**	0.875
Exercise social support	−0.428	23.053	**0.000**	0.652	−0.683	48.591	**0.000**	0.505
Fatigue	1.052	7.544	**0.006**	2.864	1.575	13.929	**0.000**	4.829

Bold values indicate statistical significance (*p* < 0.05).

## 4 Discussion

### 4.1 Current status of kinesiophobia among young and middle-aged patients with CHD

This study of 281 young and middle-aged patients with CHD revealed a mean kinesiophobia score of 50 (43, 56). The proportion of patients with moderate and high levels of kinesiophobia was 87.2%, which was consistent with the research results of Wang et al. (22). However, in this study, the proportion of patients with high-level kinesiophobia was significantly higher. We speculate that this difference might be attributed to the fact that this study focused on the specific group of young and middle-aged people. Compared with elderly patients, they often face greater pressure in career development and family responsibilities. Their concerns about the impact of sports injuries on work and family functions may be more prominent, thereby intensifying their degree of fear. A meta-analysis shows that the incidence of kinesiophobia among patients with CHD is 64% (23), characterized by irrational fear and avoidance of physical activities due to excessive worry that exercise may cause physical harm. Such fear-avoidance behaviors can lead to decreased adherence to exercise-based cardiac rehabilitation, thereby impeding the rehabilitation process and compromising its effectiveness. Therefore, it is crucial in clinical practice to routinely screen for and precisely assess kinesiophobia among young and middle-aged patients with CHD, and to develop effective intervention strategies accordingly to improve their rehabilitation adherence and clinical outcomes.

### 4.2 Heterogeneity in kinesiophobia among young and middle-aged patients with CHD

This study revealed that there is significant heterogeneity in the level of kinesiophobia among young and middle-aged patients with CHD. This study classified kinesiophobia among young and middle-aged patients with CHD into three categories. (1) Patients with “low kinesiophobia-selective sensitivity type” accounted for 12.8%. The average score of kinesiophobia item in this category is relatively low, but item 19, “My heart problem may get worse if I stay inactive” shows a specific increase, indicating that this group of patients hold a positive and optimistic attitude toward exercise and are able to participate in moderate physical training. However, patients have insufficient awareness of the importance of exercise for disease recovery and are worried that exercise will aggravate their condition. (2) The proportion of patients with “moderate kinesiophobia-perceptual sensitivity type” was 40.9%. These patients have a certain degree of danger perception of exercise-related risks and have moderate kinesiophobia. (3) The proportion of patients with “high kinesiophobia-fear avoidance type” was 46.3%. These patients have high scores in each item of kinesiophobia, are significantly restricted in exercise, lacking confidence in self-exercise, and are in a state of fear avoidance toward exercise. Therefore, in clinical practice, the psychological state and the level of kinesiophobia of patients of different categories should be evaluated in a timely manner, and personalized intervention measures should be formulated to reduce their level of kinesiophobia.

### 4.3 Influencing factors of latent profile of kinesiophobia among young and middle-aged patients with CHD

The findings of this study suggested that patients with an educational attainment of junior high school or lower are more likely to be categorized within the “high kinesiophobia-fear avoidance type.” A meta-analysis has demonstrated that educational level is a significant determinant of kinesiophobia among patients with heart disease, with lower educational attainment being correlated with increased levels of kinesiophobia (24). The health belief model (HBM) is a classic framework for health behavior decision-making, positing that behavior choice hinges on the trade-off between perceived threat (perceived susceptibility and perceived severity) and behavioral evaluation (perceived benefits and perceived barriers) (25). Patients with lower educational attainment typically have limited health literacy. They lack scientific understanding of CHD pathophysiology, exercise rehabilitation dose–response relationships, and safety boundaries, resulting in systematic cognitive biases across all four core HBM dimensions. They overestimate both the perceived severity of exercise-related risks (equating minor discomforts like tachycardia or mild chest tightness to myocardial ischemia) and their perceived susceptibility to exercise-induced heart events, while underestimating the perceived benefits of regular exercise and overestimating perceived barriers. This cognitive imbalance makes exercise avoidance their default self-protective strategy. In contrast, higher-educated patients objectively assess exercise risks and benefits, thus maintaining a rational attitude and belonging to the “low kinesiophobia-selective sensitivity type.”

In addition to sociodemographic characteristics, the severity of the condition perceived by patients is also a key factor influencing their kinesiophobia. This study found that compared with patients who had more than one stent implanted, patients with one or fewer stents were less likely to be classified as the “high kinesiophobia-fear avoidance type” and “moderate kinesiophobia-perceptual sensitivity type.” From the HBM perspective, the number of stents implanted is a key clinical determinant of patients’ disease threat perception. Multiple stents significantly elevate patients’ perceived susceptibility and perceived severity of heart events. This amplified threat perception triggers the vicious cycle described by the fear-avoidance model (FAM), which has been proven in cardiovascular care to explain fear of heart events. Patients’ exaggerated view of how severe their disease is leads to catastrophic thinking about heart problems caused by exercise. They no longer see exercise as a treatment that protects the heart, but as something that could easily cause a heart attack. Patients become extremely alert to any feelings in their body during exercise, and stop right away at the smallest sign of something wrong. Avoiding exercise for a long time makes their heart and lungs weaker and causes more physical discomfort. This makes them even more convinced that “exercise is bad for the heart,” and completes the cycle.

In contrast to the above-mentioned risk factors, a positive mental state is an important protective factor in reducing kinesiophobia. Patients with higher scores in exercise self-efficacy have a lower probability of being classified as “high kinesiophobia-fear avoidance type” and “moderate kinesiophobia-perceptual sensitivity type.” Previous studies showed that exercise self-efficacy is an important predictor of patients’ kinesiophobia (24, 26). Self-efficacy refers to an individual’s confidence in their ability to successfully complete a specific task or achieve a particular goal (27). Specifically, patients with high self-efficacy are more confident in their ability to safely complete exercise rehabilitation. They are more likely to set reasonable, gradual exercise goals and can effectively adjust themselves when experiencing mild discomfort, rather than stopping immediately and feeling fearful. In contrast, patients with low self-efficacy often have negative expectations about exercise outcomes and believe they cannot control the risks during exercise. Any physical sensation can trigger strong feelings of helplessness and fear, leading to avoidance behaviors.

Social support is another key factor in alleviating kinesiophobia. Patients with higher scores in exercise social support have a lower probability of being classified as “high kinesiophobia-fear avoidance type” and “moderate kinesiophobia-perceptual sensitivity type.” The study confirmed that a lack of social support is an important risk factor for patients’ kinesiophobia (28). The support from family, friends and medical staff can effectively cushion the psychological pressure brought by the disease, thereby reducing the level of kinesiophobia. Emotional encouragement and comfort can directly alleviate patients’ anxiety. Correct guidance in terms of information can help them correct their wrong perceptions of exercise. The successful experiences from rehabilitation peers can provide the most intuitive proof of security and directly reduce kinesiophobia.

The higher the degree of fatigue of the patients, the higher the probability of being classified as the “high kinesiophobia-fear avoidance type” and the “moderate kinesiophobia-perceptual sensitivity type,” which is consistent with the research results of Tong et al. (29). Previous research showed that 40% of patients with CHD report fatigue for more than 3 days a week, and each session lasts for more than half a day (30). Multiple studies have shown that fatigue is a factor influencing patients’ kinesiophobia (31, 32). Aaronson et al. defined fatigue as “a feeling of decline in physical and mental functions due to an imbalance in the feasibility, utilization and recoverability of energy required for the body’s activities” (33). However, patients often catastrophically misinterpret this fatigue as a sign of myocardial ischemia or cardiac insufficiency. This catastrophic cognition leads them to believe that exercise will further burden the heart and trigger dangerous events. For self-protection, patients choose to reduce or even stop physical activities. But this avoidance behavior causes a decline in physical functions, which in turn worsens fatigue. The increased fatigue then reinforces the false belief that “exercise harms the heart,” completing the vicious cycle described by the FAM.

### 4.4 Implications

#### 4.4.1 Implications for individual patient care

Clinicians should implement stratified kinesiophobia screening and differentiated interventions aligned with each profile’s core characteristics. For patients with low kinesiophobia-selective sensitivity type, interventions focus on cognitive bias correction. Clinicians should explain the dose–response relationship between exercise and cardiac safety, emphasize regular exercise’s long-term benefits in preventing recurrence, and provide a printed checklist of safe daily activities and exercise cessation warning signs. For patients with moderate kinesiophobia-perceptual sensitivity type, interventions focus on cognitive correction and gradual exposure. Clinicians should use peer success stories to deliver evidence-based risk education, demonstrate basic warm-up/cool-down techniques in person, and design personalized progression plans starting with 5–10 min of daily low-intensity walking to build confidence incrementally. For patients with high kinesiophobia-fear avoidance type, interventions focus on building safety and addressing individual barriers. Targeted measures based on key influencing factors include: using plain language and illustrated brochures for low-education patients; reviewing coronary angiography results to visually explain stent stability for those with multiple stents; setting small weekly exercise goals with timely positive reinforcement to boost self-efficacy; involving a primary family caregiver in all education sessions for patients with limited social support; and teaching energy conservation techniques for those with significant fatigue.

#### 4.4.2 Implications for rehabilitation program design

Existing cardiac rehabilitation programs should be optimized to meet the specific needs of young and middle-aged patients with CHD across care settings. In tertiary hospital cardiac rehabilitation centers, program designers should develop three distinct modules matching the three kinesiophobia profiles, with tailored starting intensities, progression rates and educational content. They should integrate structured family-based components through monthly family education workshops and caregiver training for daily exercise supervision. Establish a peer support system pairing high kinesiophobia patients with recovered peers of similar clinical backgrounds to share experiences. In primary care settings, focus on routine screening and long-term follow-up. Providers should use the Fact-CAD scale to screen for kinesiophobia during regular visits and refer high-risk patients to tertiary rehabilitation centers. Offer flexible evening/weekend sessions to accommodate work schedules, and provide remote guidance via WeChat or phone for patients unable to attend in-person visits regularly.

#### 4.4.3 Implications for policy

At the healthcare system level, policymakers should incorporate routine kinesiophobia screening using the Fact-CAD scale into standard CHD discharge assessments to identify and refer high-risk patients. Expand medical insurance coverage for cardiac rehabilitation to reduce financial barriers, particularly for young and middle-aged patients who are primary family earners. Strengthen training for primary care providers in kinesiophobia assessment and intervention, and establish a hierarchical referral system between tertiary hospitals and community health centers for continuous care.

## 5 Strengths and limitations

The innovation of this study lay in employing LPA to identify latent categories of kinesiophobia among young and middle-aged patients with CHD, while also exploring the influencing factors across different categories. This not only revealed significant heterogeneity in kinesiophobia within the patient population but also provided empirical evidence for developing precise, personalized intervention strategies. However, this study has several limitations that should be noted when interpreting the findings. First, this study was conducted at a single center, and all included patients were from the same region with similar socioeconomic status and healthcare resource accessibility. Thus, the generalizability of the identified three kinesiophobia latent profiles and their influencing factors to other regions, primary medical institutions, and patients with different economic levels may be limited. Second, this study adopted a cross-sectional design, which can only identify the correlation between influencing factors and kinesiophobia profiles, but cannot verify the causal relationship between variables. Therefore, the findings cannot directly support the causal inference of targeted intervention, and further longitudinal cohort studies are needed to confirm the dynamic changes of kinesiophobia profiles and the causal effect of influencing factors. Third, cultural and healthcare system differences must be taken into account when extrapolating our findings to other countries. These cultural and systemic differences may affect the effectiveness of implementing our proposed intervention strategies in other contexts.

## 6 Conclusion

This study showed that kinesiophobia among young and middle-aged patients with CHD was a moderate to high level. Kinesiophobia can be classified into three categories, namely: low kinesiophobia-selective sensitivity type, moderate kinesiophobia-perceptual sensitivity type, high kinesiophobia-fear avoidance type. Different educational attainment, the number of stents, exercise self-efficacy, exercise social support and fatigue were significantly correlated with the level of kinesiophobia.

## Data Availability

The original contributions presented in the study are included in the article/Supplementary material, further inquiries can be directed to the corresponding author.
