# Supplementary material for: Latent profile analysis and influencing factors of kinesiophobia among young and middle-aged patients with coronary heart disease
Source: Front Med (Lausanne). 2026 May 13;13:1795966. doi: 10.3389/fmed.2026.1795966 (PMC13212131; doi:10.3389/fmed.2026.1795966)
Supplement: Supplementary file 1 [file Supplementary_file_1.docx]

**Supplementary 1.**

Fact-CAD Scale Description

The Fear of Activity in Patients with Coronary Artery Disease (Fact-CAD) scale is a 21‑item self‑report instrument. Each item is rated on a 5‑point Likert scale (0 = never, 1 = seldom, 2 = sometimes, 3 = mostly, 4 = always). Seven items (Items 1, 5, 9, 13, 17, 19, 21) are positively worded and are inversely scored (0 = always, 1 = mostly, 2 = sometimes, 3 = seldom, 4 = never). The total score ranges from 0 to 84, with higher scores indicating greater fear of activity.

For the complete scale items, scoring instructions, and validation details, please refer to the original publication:

Ozyemisci-Taskiran O, Demirsoy N, Atan T, et al. Development and Validation of a Scale to Measure Fear of Activity in Patients With Coronary Artery Disease (Fact-CAD). *Arch Phys Med Rehabil*. 2020;101(3):479-486. doi:10.1016/j.apmr.2019.09.001

Note: Due to copyright considerations, the original scale text is not reproduced here. Readers are directed to the original source for the full scale.
